# Supplementary material for: Predator gaze captures both human and chimpanzee attention
Source: PLoS One. 2024 Nov 21;19(11):e0311673. doi: 10.1371/journal.pone.0311673 (PMC11581262; doi:10.1371/journal.pone.0311673)
Supplement: S1 File — (DOCX) [file pone.0311673.s001.docx]

**Supplement for:**

Predator gaze captures both human and chimpanzee attention

**Validation procedure**

To confirm that the calibration procedures resulted in accurate tracking of chimpanzee gaze, we performed validation procedures after each calibration and before beginning each experimental session. First, an onscreen target (a video similar to those used for the calibration) appeared at one of eight onscreen locations that roughly spanned the space that experimental stimuli would occupy onscreen. After the chimpanzee visually fixated in the vicinity of this validation point, they received a juice reward and the target moved to another of the eight onscreen locations. The full procedure took less than a few minutes.

To estimate whether chimpanzee fixations on these validation points was accurate, we used the standardized procedure suggested by Kar and Corcoran (2018) for eye-tracker evaluation. This procedure is independent of any specific hardware or data cleaning algorithm. Briefly, for each validation point location:

1. Mean X and Y gaze point coordinates, in pixels, were computed by averaging the raw coordinates of left eye and right eye gaze data across the samples directed to the validation point:

$$GazeX=mean\left( \frac{X_{left}+X_{right}}{2} \right), GazeY=mean\left( \frac{Y_{left}+Y_{right}}{2} \right)$$

Equation 1

1. On-screen distance (OSD) between each mean gaze coordinate and the center of the screen was computed by using the Euclidean distance formula to derive the distance, in pixels, between the points, then scaling these values to mm of on screen distance by multiplying them by the pixel size (*µ*) of the Tobii Tx300 Pro monitor (~.26 mm):

$$OSD= \mu\sqrt{\left( GazeX-\frac{screen\_width}{2} \right)^{2}+ \left( GazeY-\frac{screen\_height}{2} \right)^{2}}$$

Equation 2

1. The gaze angle (θ) of an onscreen point relative to the chimpanzee’s eye suggested by these pixel distances was computed using trigonometry and an estimate of the distance from the chimpanzee eye to the screen (Z = 630 mm):

$$\theta= {tan}^{-1}\left( OSD/Z \right)$$

Equation 3

1. The distance between eye and gaze point (EGP) was computed using 3D Cartesian geometry:

$$EGP= \sqrt{{GazeX}^{2}+ {GazeY}^{2}+ Z^{2}}$$

Equation 4

1. The pixel distance (pix_dist) between gaze coordinates and the center of validation points was computed using the Euclidean distance formula:

$$pix\_dist = \sqrt{\left( ValidationX-GazeX \right)^{2}+\left( ValidationY-GazeY \right)^{2}}$$

Equation 5

1. Finally, the values derived in the above equations were combined into a single measure, angular accuracy (in degrees), that captures the deviation between observed and expected gaze:

$$AngularAccuracy=\left( \mu*pix\_dist*{cos\left( mean\left( \theta\right) \right)}^{2} \right)/EGP$$

Equation 6

For more details and equations on these procedures, refer to the original publication (Kar & Corcoran, 2018). Figure S1 displays an example of a typical validation outcome from a chimpanzee. Based on this validation procedure, the accuracy of the eye-tracker using chimpanzee subjects was approximately 1.5 degrees.


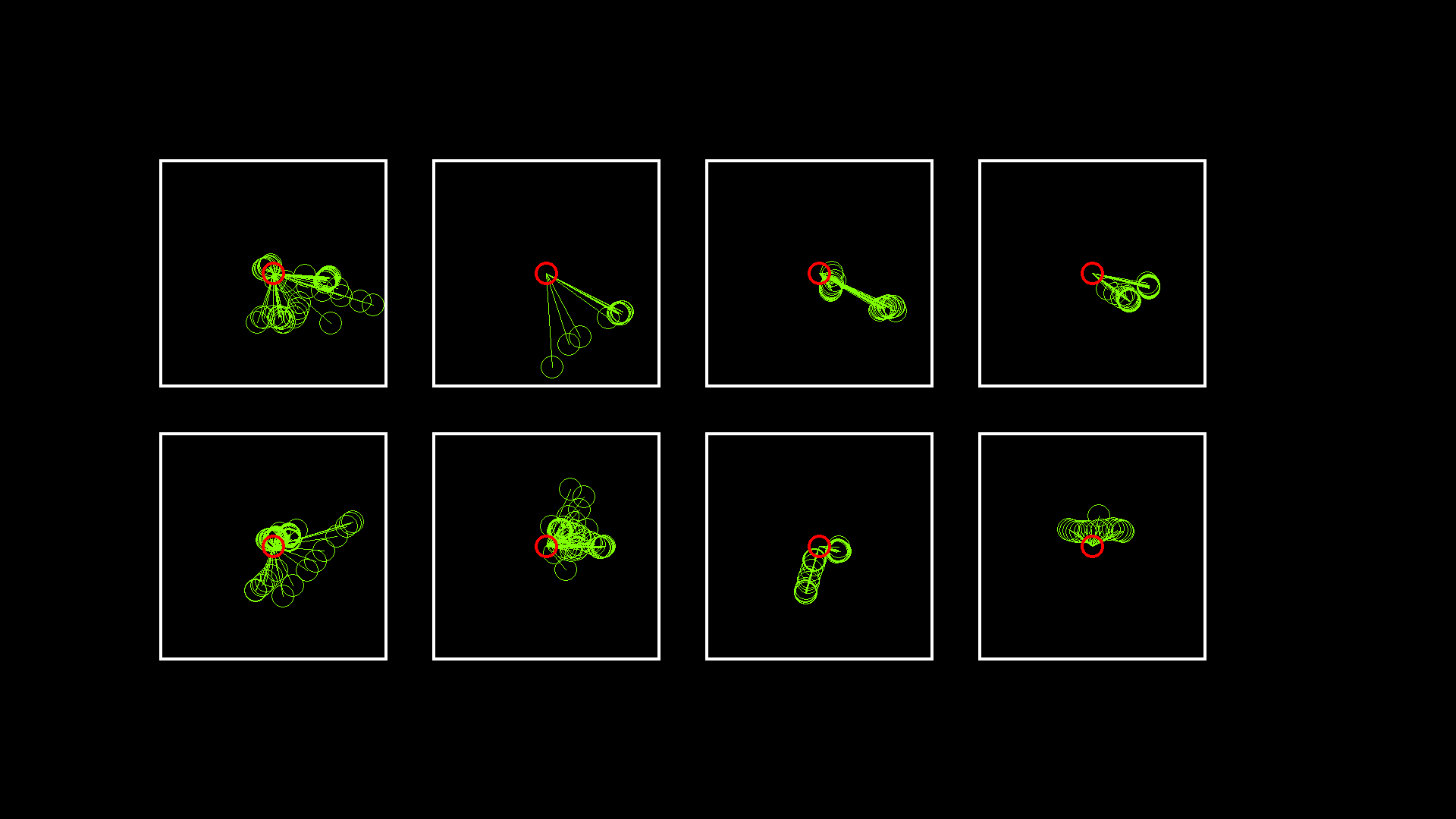


Figure S1. Output of the validation procedure. The open circles represent the observed gaze locations while the red dots are the center of each validation point (i.e., the expected gaze location).

It is important to note that the chimpanzees were not necessarily fixating directly on the center of the validation points. The angular accuracy therefore includes sources of error other than eye-tracker miscalibration. That the chimpanzee angular accuracies were nevertheless as low as they were suggest that the eye-tracker quickly and accurately detected chimpanzee gaze across the entire screen.

References

Kar, A., & Corcoran, P. (2018). Performance evaluation strategies for eye gaze estimation systems with quantitative metrics and visualizations. Sensors (Switzerland), 18(9). https://doi.org/10.3390/s18093151
