# Supplementary material for: Predator gaze captures both human and chimpanzee attention
Source: PLoS One. 2024 Nov 21;19(11):e0311673. doi: 10.1371/journal.pone.0311673 (PMC11581262; doi:10.1371/journal.pone.0311673)
Supplement: S1 Table — (DOCX) [file pone.0311673.s002.docx]

**Supplement for:**

Predator gaze captures both human and chimpanzee attention

**S2 Table. *p* values for *post hoc* comparison matrix for Experiment 1**

|  | **I-A-Bk** | **I-A-By** | **I-A-E** | **I-A-H** | **I-D-Bk** | **I-D-By** | **I-D-E** | **I-D-H** | **L-A-Bk** | **L-A-By** | **L-A-E** | **L-A-H** | **L-D-Bk** | **L-D-By** | **L-D-E** | **L-D-H** |
| --- | --- | --- | --- | --- | --- | --- | --- | --- | --- | --- | --- | --- | --- | --- | --- | --- |
| **I-A-Bk** | 1.000 | <.001 | <.001 | <.001 | 1.000 | <.001 | <.001 | <.001 | 0.002 | <.001 | <.001 | <.001 | 0.003 | <.001 | <.001 | <.001 |
| **I-A-By** | <.001 | 1.000 | <.001 | <.001 | <.001 | 1.000 | <.001 | <.001 | <.001 | 1.000 | <.001 | <.001 | <.001 | <.001 | <.001 | <.001 |
| **I-A-E** | <.001 | <.001 | 1.000 | <.001 | <.001 | <.001 | 1.000 | <.001 | <.001 | <.001 | 0.293 | <.001 | <.001 | <.001 | <.001 | <.001 |
| **I-A-H** | <.001 | <.001 | <.001 | 1.000 | <.001 | <.001 | <.001 | 0.019 | <.001 | <.001 | <.001 | 0.956 | <.001 | <.001 | <.001 | <.001 |
| **I-D-Bk** | 1.000 | 0.000 | <.001 | <.001 | 1.000 | <.001 | <.001 | <.001 | <.001 | <.001 | <.001 | <.001 | <.001 | <.001 | <.001 | <.001 |
| **I-D-By** | <.001 | 1.000 | <.001 | <.001 | <.001 | 1.000 | <.001 | <.001 | <.001 | 1.000 | <.001 | <.001 | <.001 | <.001 | <.001 | <.001 |
| **I-D-E** | <.001 | <.001 | 1.000 | <.001 | <.001 | <.001 | 1.000 | <.001 | <.001 | <.001 | <.001 | <.001 | <.001 | <.001 | <.001 | <.001 |
| **I-D-H** | <.001 | <.001 | <.001 | 0.019 | <.001 | <.001 | <.001 | 1.000 | <.001 | <.001 | <.001 | <.001 | <.001 | <.001 | <.001 | <.001 |
| **L-A-Bk** | 0.002 | <.001 | <.001 | <.001 | <.001 | <.001 | <.001 | <.001 | 1.000 | <.001 | <.001 | <.001 | 1.000 | <.001 | <.001 | <.001 |
| **L-A-By** | <.001 | 1.000 | <.001 | <.001 | <.001 | 1.000 | <.001 | <.001 | <.001 | 1.000 | <.001 | <.001 | <.001 | <.001 | <.001 | <.001 |
| **L-A-E** | <.001 | <.001 | 0.293 | <.001 | <.001 | <.001 | <.001 | <.001 | <.001 | <.001 | 1.000 | <.001 | <.001 | <.001 | <.001 | <.001 |
| **L-A-H** | <.001 | <.001 | <.001 | 0.956 | <.001 | <.001 | <.001 | <.001 | <.001 | <.001 | <.001 | 1.000 | <.001 | <.001 | <.001 | <.001 |
| **L-D-Bk** | 0.003 | <.001 | <.001 | <.001 | <.001 | <.001 | <.001 | <.001 | 1.000 | <.001 | <.001 | <.001 | 1.000 | <.001 | <.001 | <.001 |
| **L-D-By** | <.001 | <.001 | <.001 | <.001 | <.001 | <.001 | <.001 | <.001 | <.001 | <.001 | <.001 | <.001 | <.001 | 1.000 | <.001 | <.001 |
| **L-D-E** | <.001 | <.001 | <.001 | <.001 | <.001 | <.001 | <.001 | <.001 | <.001 | <.001 | <.001 | <.001 | <.001 | <.001 | 1.000 | <.001 |
| **L-D-H** | <.001 | <.001 | <.001 | <.001 | <.001 | <.001 | <.001 | <.001 | <.001 | <.001 | <.001 | <.001 | <.001 | <.001 | <.001 | 1.000 |

I=Impala, L=Lion, A=Averted, D=Directed, Bk=Background, By=Body, E=Eyes, H=Head
