# Supplementary material for: Predator gaze captures both human and chimpanzee attention
Source: PLoS One. 2024 Nov 21;19(11):e0311673. doi: 10.1371/journal.pone.0311673 (PMC11581262; doi:10.1371/journal.pone.0311673)
Supplement: S2 Table — (DOCX) [file pone.0311673.s003.docx]

**Supplement for:**

Predator gaze captures both human and chimpanzee attention

**S8 Table. *p* values for *post hoc* comparison matrix for Experiment 2**

|  | **I-A-Bk** | **I-A-By** | **I-A-E** | **I-A-H** | **I-D-Bk** | **I-D-By** | **I-D-E** | **I-D-H** | **L-A-Bk** | **L-A-By** | **L-A-E** | **L-A-H** | **L-D-Bk** | **L-D-By** | **L-D-E** | **L-D-H** |
| --- | --- | --- | --- | --- | --- | --- | --- | --- | --- | --- | --- | --- | --- | --- | --- | --- |
| **I-A-Bk** | 1.000 | <.001 | 1.000 | 0.731 | 1.000 | <.001 | 1.000 | 1.000 | 0.086 | <.001 | 0.141 | <.001 | <.001 | 1.000 | <.001 | <.001 |
| **I-A-By** | <.001 | 1.000 | <.001 | <.001 | <.001 | 1.000 | <.001 | <.001 | <.001 | <.001 | <.001 | 0.001 | <.001 | <.001 | 1.000 | <.001 |
| **I-A-E** | 1.000 | <.001 | 1.000 | 1.000 | 1.000 | <.001 | 1.000 | 1.000 | 1.000 | <.001 | 1.000 | <.001 | 0.008 | 1.000 | <.001 | <.001 |
| **I-A-H** | 0.731 | <.001 | 1.000 | 1.000 | 1.000 | <.001 | 1.000 | 1.000 | <.001 | 0.028 | 1.000 | <.001 | <.001 | 1.000 | <.001 | 0.027 |
| **I-D-Bk** | 1.000 | <.001 | 1.000 | 1.000 | 1.000 | <.001 | 1.000 | 1.000 | 0.001 | <.001 | 1.000 | <.001 | <.001 | 1.000 | <.001 | <.001 |
| **I-D-By** | <.001 | 1.000 | <.001 | <.001 | <.001 | 1.000 | <.001 | <.001 | <.001 | <.001 | <.001 | 0.001 | <.001 | <.001 | 1.000 | <.001 |
| **I-D-E** | 1.000 | <.001 | 1.000 | 1.000 | 1.000 | <.001 | 1.000 | 1.000 | 0.242 | 0.001 | 1.000 | <.001 | 0.001 | 1.000 | <.001 | 0.001 |
| **I-D-H** | 1.000 | <.001 | 1.000 | 1.000 | 1.000 | <.001 | 1.000 | 1.000 | 0.023 | <.001 | 1.000 | <.001 | <.001 | 1.000 | <.001 | <.001 |
| **L-A-Bk** | 0.086 | <.001 | 1.000 | <.001 | 0.001 | <.001 | 0.242 | 0.023 | 1.000 | <.001 | <.001 | <.001 | 0.101 | <.001 | <.001 | <.001 |
| **L-A-By** | <.001 | <.001 | <.001 | 0.028 | <.001 | <.001 | 0.001 | <.001 | <.001 | 1.000 | 0.837 | 1.000 | <.001 | <.001 | 0.034 | 1.000 |
| **L-A-E** | 0.141 | <.001 | 1.000 | 1.000 | 1.000 | <.001 | 1.000 | 1.000 | <.001 | 0.837 | 1.000 | 0.034 | <.001 | 1.000 | <.001 | 0.863 |
| **L-A-H** | <.001 | 0.001 | <.001 | <.001 | <.001 | 0.001 | <.001 | <.001 | <.001 | 1.000 | 0.034 | 1.000 | <.001 | <.001 | 0.713 | 1.000 |
| **L-D-Bk** | <.001 | <.001 | 0.008 | <.001 | <.001 | <.001 | 0.001 | <.001 | 0.101 | <.001 | <.001 | <.001 | 1.000 | <.001 | <.001 | <.001 |
| **L-D-By** | 1.000 | <.001 | 1.000 | 1.000 | 1.000 | <.001 | 1.000 | 1.000 | <.001 | <.001 | 1.000 | <.001 | <.001 | 1.000 | <.001 | <.001 |
| **L-D-E** | <.001 | 1.000 | <.001 | <.001 | <.001 | 1.000 | <.001 | <.001 | <.001 | 0.034 | <.001 | 0.713 | <.001 | <.001 | 1.000 | 0.025 |
| **L-D-H** | <.001 | <.001 | <.001 | 0.027 | <.001 | <.001 | 0.001 | <.001 | <.001 | 1.000 | 0.863 | 1.000 | <.001 | <.001 | 0.025 | 1.000 |

I=Impala, L=Lion, A=Averted, D=Directed, Bk=Background, By=Body, E=Eyes, H=Head
