# Supplementary material for: Predator gaze captures both human and chimpanzee attention
Source: PLoS One. 2024 Nov 21;19(11):e0311673. doi: 10.1371/journal.pone.0311673 (PMC11581262; doi:10.1371/journal.pone.0311673)
Supplement: S1 Fig — (DOCX) [file pone.0311673.s004.docx]

**Supplement for:**

Predator gaze captures both human and chimpanzee attention

**S1 Fig. *Post hoc* comparison matrix for Experiment 1**

| 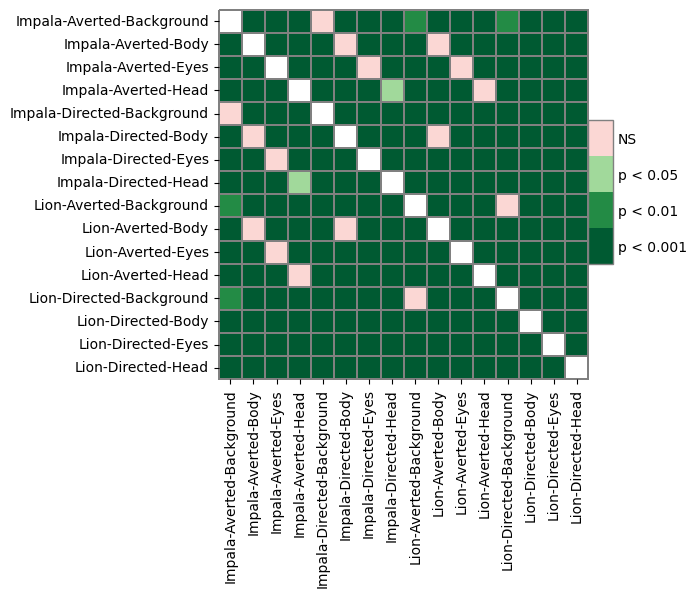 |
| --- |
