# Supplementary material for: Predator gaze captures both human and chimpanzee attention
Source: PLoS One. 2024 Nov 21;19(11):e0311673. doi: 10.1371/journal.pone.0311673 (PMC11581262; doi:10.1371/journal.pone.0311673)
Supplement: S2 Fig — (A) the eye-tracker and all associated hardware positioned on a rolling cart approximately 63 cm from the chimpanzee mesh, and (B) an overhead view of a chimpanzee participating in the experiment. Photograph taken by WW and printed under a CC BY license. (DOCX) [file pone.0311673.s005.docx]

**Supplement for:**

Predator gaze captures both human and chimpanzee attention

**Figure S3. Photographs of the experimental setup.** (A) the eye-tracker and all associated hardware positioned on a rolling cart approximately 63 cm from the chimpanzee mesh, and (B) an overhead view of a chimpanzee participating in the experiment.

| A  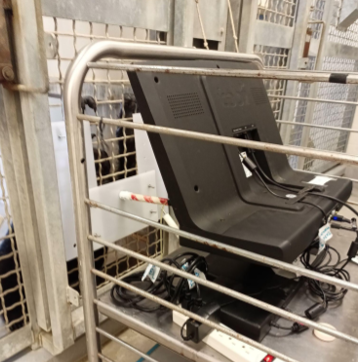 | B  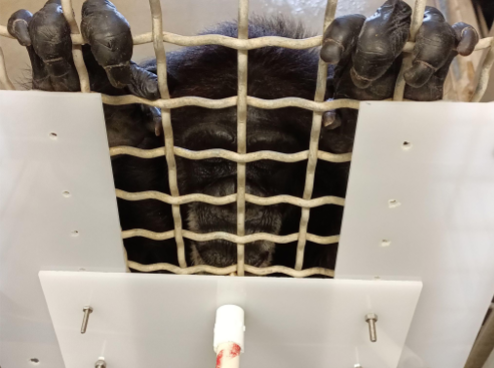 |
| --- | --- |
