# Supplementary material for: Predator gaze captures both human and chimpanzee attention
Source: PLoS One. 2024 Nov 21;19(11):e0311673. doi: 10.1371/journal.pone.0311673 (PMC11581262; doi:10.1371/journal.pone.0311673)
Supplement: S4 Fig — Fixation duration plotted as a function of stimulus species, gaze direction and ROI for chimpanzee subjects. (DOCX) [file pone.0311673.s007.docx]

**Supplement for:**

Predator gaze captures both human and chimpanzee attention

**S6 Supplemental analyses. Fixation durations plotted as a function of stimulus type, gaze direction and ROI for chimpanzee subjects.**

| 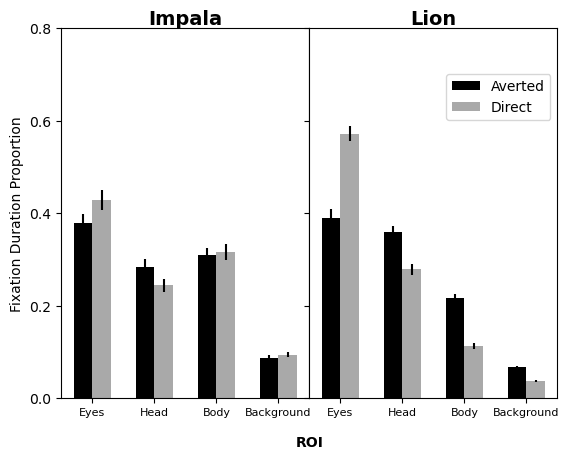 |
| --- |

| **Overall Model** |  |  |  |
| --- | --- | --- | --- |
|  | **Factor** | ***F_df,df error_*** | ***p*** |
|  | Stimulus species | 8.98_1,6_ | .02* |
|  | Gaze direction | 0_1,6_ | 0.957 |
|  | ROI | 45.61_3, 18_ | <.001* |
|  | Stimulus species* Gaze direction | 2.90_1,6_ | .139 |
|  | Stimulus species* ROI | 43.10_3, 18_ | <.001* |
|  | Gaze direction*ROI | 8.20_3, 18_ | .001* |
|  | Stimulus species* Gaze direction* ROI | 4.29_3, 18_ | .019* |
| **Comparisons** |  |  |  |
|  | Lion |  |  |
|  | Eyes Directed vs. Eyes Averted |  | .004* |
|  | Head Directed vs. Head Averted |  | <.001* |
|  | Body Directed vs. Body Averted |  | .007* |
|  | Eyes vs. Head |  | <.001* |
|  | Eyes vs. Body |  | <.001* |
|  | Eyes vs. Background |  | <.001* |
|  | Impala |  |  |
|  | Eyes Directed vs. Eyes Averted |  | .583 |
|  | Head Directed vs. Head Averted |  | .577 |
|  | Body Directed vs. Body Averted |  | .888 |
|  | Eyes vs. Head |  | .385 |
|  | Eyes vs. Body |  | <.001* |
|  | Eyes vs. Background |  | <.001* |
|  | Eyes |  |  |
|  | Lion vs. Impala |  | .116 |
|  | Head |  |  |
|  | Lion vs. Impala |  | <.001* |
|  | Body |  |  |
|  | Lion vs. Impala |  | .501 |
