# Supplementary material for: Predator gaze captures both human and chimpanzee attention
Source: PLoS One. 2024 Nov 21;19(11):e0311673. doi: 10.1371/journal.pone.0311673 (PMC11581262; doi:10.1371/journal.pone.0311673)
Supplement: Attachment — Submitted filename: Response to Reviewers.docx [file pone.0311673.s009.docx]

**Reviewer #1 comments**

**R1C1 ::** This is an interesting and well-written manuscript about a topic that has not attracted much prior research and as such is welcomed. There were however some important deficiencies in theoretical justification and experimental design (or insufficient explanation) that need to be addressed. Perhaps the authors have already thought through and solved many of these, but the reasoning should be included in the manuscript for readers to follow all points of their logic.

Response :: We thank the reviewer for their constructive review of the manuscript and for giving us the opportunity to present a more clearly reasoned, easier-to-follow manuscript.

Major concerns:
**R1C2 ::** The paper refers throughout to predator and prey gaze, but tests only lion and impala with no other species. This seriously limits the generalizability of their conclusions. This is compounded by fairly weak justification of species choice. It is true lions co-occur with eastern chimpanzees in some narrow savannah regions where chimpanzees are likely hunted to at least some extent, but most captive chimpanzees, and presumably the current population, are western chimpanzees. Beyond subspecies differences, most chimpanzees live in forest environments. For both of these reasons, leopards are much more ecologically relevant and indeed have been found to predate chimpanzees across their range more than has been reported for lions. Further, the stimuli examples appeared to show male lions, who do not typically engage in much of the hunting, and lions further are group-hunters (not solitary as are leopards) and only one individual at a time was presented. While the big cat morphology may be a form that is relatively conserved, and predates upon many primates across the world including humans and chimpanzees, the text only makes a very vague association with one species in one part of a different subspecies’ range. I recommend justifying this point in the text, that we may expect conserved attentional biases to come through across big cat morphologies including the lions used in this study. Relatedly, on the other side, chimpanzees are proficient hunters themselves but do not hunt impala. Duikers, squirrels, red colobus monkeys, would all be more species-relevant prey to a chimpanzee, why was impala chosen? This should also be mentioned when discussing why human attention may differ from other species. Chimpanzees, like humans, are both predators are prey to different animals, but the stimuli only included species that are predator and prey to humans. It is thus not really a fair species comparison. The discussion certainly should mention the lack of species diversity as a limitation on generalizability and future direction, even moreso in light of these larger concerns.

Response :: We agree that the language of our original submission both obfuscated our intent and suggested a set of ecological/evolutionary inquiries that our experiments could not effectively test. We have clarified throughout (ll 140-164; 165-171) that our questions, hypotheses, and experiments were targeted to gaze behaviors related to the prototypical features of predators (e.g., forward-facing eyes, robust body) and prey (e.g., lateral eyes) exemplified by the lion and impala. We make no claims about primate behavioral ecology except as related to any deeply conserved attentional biases that ancestral primate evolutionary history may have introduced. The lack of diversity in our sample of predator and prey images is indeed a sincere limitation (albeit an intentional one, designed to measure attentional biases in limited time with a highly unique chimpanzee population) and an appropriate target for future studies. We have added language about this limitation to the discussion (ll 474-478).

**R1C3 ::** The inclusion of the saliency maps felt a little out of place and I did not find them integrated into the rest of the discussion. Some introductions of their actual use and predictive power in the introduction would be of use. As of now, they are introduced only in ways they fail to predict gaze, and then the results again fail to find them making new predictions, and the naïve reader is left wondering what these can contribute if they consistently fail to make predictions.

Response :: We have added a paragraph to the introduction (ll 181-194) to justify the inclusion of salience maps to test the conservative (and arguably default) alternative hypothesis that any differences in gaze behavior can be understood, with fewer assumptions, to be the result of low-level stimulus features. We believe that such tests are a prerequisite for making more complex arguments from cognitive representation and primate evolutionary biology that our predictions require.

**R1C4 ::** The hypotheses are quite vague and not well-justified, which combined with a very high number of statistical tests leads readers to wonder if this was more exploratory or hypothesis-driven. Both are welcomed, especially with new topics such as this, but perhaps the manuscript could benefit from distinguishing them and making clearer, concrete predictions associated with the actual hypotheses (ideally with citation). In particular, the first set of predictions (paragraph starting line 131) is essentially just sameness or difference, but then no statistical tests actually directly compared the two species. The lack of direct human-chimpanzee comparison is a strange choice. The reasoning of the sentence starting on line 135 is especially vague and nebulous. If these were the topics of interest wouldn’t a study comparing humans with different background knowledge and social contexts be a more salient test? The choice of a cross-species framework here could be much better-justified.

Response :: We acknowledge that the original formulation of our predictions was not always clear, and we have substantially edited these sections referenced above to better convey the intent of our experiments (ll 140-164). We tested the same set of predictions – about eye direction, prototypical predator and prey features, etc – in both humans and chimpanzees with an eye towards demonstrating which primate attentional biases may have been maintained over evolutionary time. We have clarified that we do not expect differences among the humans and chimpanzees’ gaze behaviors, as these would suggest some outsized influence of background knowledge, sociocultural forces, learning, or the other enculturating forces Reviewer 2 identified (“humans have arguably different relationships with lions/impalas than (captive) chimpanzees. For example, lions are typically considered charismatic species that are highly favoured to see in zoos or safaris.”), which we did not predict nor intend to test (ll 140-152). The nature of these human-chimpanzee hypotheses is a poor fit for statistical tests since the parsimonious, literature-informed hypothesis is the null, which we cannot test, since our predictions are primarily about which species/regions are fixated most frequently (not their absolute proportion or rank order), and since any effects are nested in complex interactions with all of the other factors we tested.

**R1C5 ::** The dependent measure as proportion of fixation is very unusual compared to previous great ape work. Why was total fixation duration not included? What reasoning is there for choosing number of fixations over duration, especially considering prior work consistently showing chimpanzee gaze is likely to jump around more than humans. Could the lion have evoked more glances away (chimpanzees tend to avoid prolonged direct eye contact) while the averted gaze and impala had longer but more consistent gaze? At the very least, supplemental analysis detailing the duration should be included somewhere.

Response :: Our dataset is indeed well-suited to many analytical frameworks. Our choice of dependent measure, over other correlated measures, is aligned with the human work published using area-normalized fixation proportions (Birmingham et al., 2008a, 2008b). We acknowledge that fixation duration and fixation number are slightly different measures, but generally strongly correlated. No new effects emerge from our dataset when the dependent measure is changed to area normalized total fixation duration, now featured as a supplement figure 4.

Minor concerns:
**R1C6 ::** Line 87: The topic of chimpanzees seems to come out of nowhere here. As they are central to the whole paper, their introduction should be naturally connected. Perhaps a linking phrase of paragraph about the value and perspective around comparative gaze studies of chimpanzees would help improve this and address one of the major comments above?

Response :: The structure and language of the introduction, especially with regard to the species under study, has been updated for clarity throughout. Several additional primate eye-tracking designs have been added or elaborated upon in this revision (ll 56-57; 66-73; 91; 93-95).

**R1C7 ::** Line 87: This finding itself also needs more rigour if chimpanzee attention to faces is a central focus of the paper. The previous studies find that chimpanzees gaze more at mouth than eyes even within faces, and this has been considered as potentially more species-relevant for social cues than eyes (Kano et al. 2018, Brooks et al 2021). Did the authors consider looking at attention to mouth by species given this finding? At the very least, the difference in the role and meaning of eye-contact across species should be mentioned. Note also that the supplemental material of some of these previous face-based studies have looked at attention to direct compared to averted gaze, which while not directly on the topic of predator/prey gaze may be useful for the authors to look at.

Response :: We have added language on chimpanzees’ relative interest in other face regions besides eyes (ll 90-92). As emphasized by reviewer 2, we are sensitive to the idea that mechanisms of social gaze to conspecifics are distinct from mechanisms of gaze to potentially dangerous heterospecifics, with the latter the target of our research with humans and chimpanzees.

**R1C8 ::** Line 116: This is a bit of a strange citation here, as the point of the sentence is that eyes have high saliency in the face but the cited paper is on the topic of that not being at all universal, and certainly is not a citation about them being “presented in this manner.”

Response :: We have clarified our use of this citation.

**R1C9 ::** Line 139: This whole paragraph does not distinguish species for the predictions. Maybe the authors intend these predictions to apply to all participants, but this should then be made explicit.

Response :: As above, we have substantially edited the way we present our predictions to clarify what we were (and were not) testing (ll 141-169).

**R1C10 ::** Line 161: The ethnicity states Chinese, Korean, and east Asian as distinct, please check this or clarify.

Response :: This was the language of the demographic survey.

**R1C11 ::** Throughout: “other part of the head” may be clearer than just head when distinguishing from eyes, since in some sentences this could easily be lost by readers.

Response :: To avoid confusion we have language in the methods pointing the reader to Figure 1B for a visualization of what is meant by eyes, head, body, and background.

**R1C12 ::** Several times: It may be a bit inaccurate to say we couldn’t look at lion-impala differences in humans because of high attention to both. It would be more accurate to say no differences were observed because of ceiling effects, or the data was not suited for a formal model because of ceiling effects but visually appeared identical. Saying both were at ceiling is a comparison itself.

Response :: We thank you for this statistical point, and have corrected our language (ll 405-406).

**R1C13 ::** Line 430: “To our knowledge, this experiment provides some of the first evidence examining the strength of the bias towards visually fixating the eyes of nonhuman animals in both human and chimpanzee participants.” This sentence must be rephrased or removed. The prior sentence cites Kano et al. 2018, which compared several primate species including humans on social attention. Also, the nonhuman animals point is somewhat missed here if talking about nonhuman participants, maybe allospecific would be more relevant.

Response :: We have rephrased this point to make clearer that what we find novel is the measured strength of bias to lion and impala eyes, rather than the kinds of within-species / within-primate comparisons that are already part of the cited literature (ll 492-494).

**R1C14 ::** General: the size of the eye ROIs appears very small in the figure, especially considering the screen size used. Are these within the margin of error of accuracy? This should be mentioned.

Response :: We have now noted that all the ROIs were within the error accuracy of the trackers (ll 352-353).

**R1C15 ::** Line 443: Chimpanzees are one of the nearest relatives, bonobos are equally closely related. Bonobos generally look much more to eyes than chimpanzees (Kano et al. 2018) and have no lions in their range (but do also face leopard predation) and are less proficient hunters. Perhaps a future comparative approach can be highlighted as a future direction?

Response :: We have added this suggestion for a target of future research (ll 531-534).

**R1C16 ::** Line 444: check divergence dates.

Response :: We have removed this extraneous language.

**R1C17 ::** General: the discussion could use more interpretation and connection to the broader literature, as it stands it is hard to see what the importance beyond restatement of the results.

Response :: We have revised the Discussion to better highlight what is novel about our results (ll 516-517), how our results were unlikely to be the product of forces other than evolved attention biases (ll 509-511; 518-520), and how future designs could further explore this topic (ll 474-478; 531-534).

**R1C18 ::** One point not raised anywhere in the manuscript that certainly bears discussion: predators have more forward-facing eyes than prey. Could you rule out the fact that there is a general direct eye-gaze bias, but that the lions just have more direct gaze (at least from the view of primates such as ourselves with forward-facing eyes) and it is therefore not related to predator/prey distinctions per se?

Response :: This is an important point, and indeed something we are very interested in. We are much more explicit in the revision that exactly these differences – between a prototypical predator phenotype and a prototypical prey phenotype – are likely determinants of differences in patterns of gaze behavior across images (ll 140-142; 154-157). Future research could clarify what features of our prototypical predator and prey are most responsible for gaze behavior biases in human and nonhuman primate attention to a much more diverse set of stimuli (ll 474-478).

**R1C19 ::** How do you account for the fact that the relative eye size between the two species is different? The eyes of the impala appear to be a much bigger proportion of the head than lions. Perhaps this strengthens the finding, but it bears discussion.

Response :: The area-normalized fixation proportions that we used as the dependent measure in all analyses scale fixation counts to a region-of-interest by the proportion of the screen that the region-of-interest occupies. The relative size of the eye region was not necessarily larger for either or lion images or our impala images.

**R1C20 ::** It is rare in eye-tracking with great apes to use a mesh rather than transparent barrier between eyes and screens. Can you be sure this did not affect gaze tracking when one or the other eye is blocked to the sensor?

Response :: The mesh was an unfortunate requirement of our access to the chimpanzees. We are nevertheless confident in our methods. Modern fixation filtering algorithms like the I2MC algorithm we used are quite good at identifying fixations in spite of momentary loss of contact with one or both eyes (please see description of the I2MC filter in ll 279-282). In pilot testing with human experimenters, mesh outperformed acrylic barriers (that can be dirty, scratched, reflective, etc). We have no reason to suspect that mesh could introduce any structural bias that could have affected our hypotheses, e.g., that noisier gaze coordinates biased analyses to lion eyes but impala backgrounds.

**R1C21 ::** Similarly, juice only being delivered while gaze is onscreen seems very odd. How can you be sure their behaviour was not simply shaped by perceived rewarding of certain gaze? Generally, juice is used to motivate them to join experiments, but rewards that are differentially delivered according to gaze is a slippery slope if aiming to target spontaneous gaze patterns.

Response :: The legacy of “superstitious” behavior in captive animals under reward schedules is indeed something to be understood and acknowledged. For our chimpanzee subjects, tested in their enriched home enclosures alongside conspecifics, husbandry staff, other experimenters, and a highly enriched captive setting, we felt again that an unfortunate concession of gaze-contingent juice reward was made as a requirement of our access to the chimpanzees. We are nevertheless confident that rewarding every on-screen gaze behavior did not introduce a bias that only applied to specific gaze behaviors in a way that immediate post-trial rewards somehow do not. We argue that reward of all gaze behavior would not incentivize similar patterns of gaze behavior in each of our chimpanzee subjects, nor patterns of gaze that broadly accord with human gaze behavior and our predictions, nor would they be maintained in the same way across very high trial counts for ape eye-tracking research, nor would they, as above, bias gaze behavior to lion eyes but impala backgrounds. We have added text to this effect to the Methods section (ll 306-311).

**R1C22 ::** How did attention change across/within trials? The trials seem longer than typical in chimpanzee research, was there any obvious effect of presentation order?

Response :: We found no effects of presentation order, and consider the relatively large dataset of chimpanzee gaze behavior a strength of our design.

**R1C23 ::** Was there consistency across individuals tested within species? This would strengthen the finding considerably given the low sample size.

Response :: We have added language confirming consistency across individuals for the central points of the manuscript (ll 424-425) and a representative supplemental figure S3.

**R1C24 ::** There are several citation errors. Ensure the reference section matches the main text (e.g. Carter et al. wrong year, Kano et al. 2018 not in reference section).

Response :: We apologize for the errors, which have now been corrected.

**Reviewer #2**

GENERAL

**R2C1 ::** In this study, Karstadt & Whitham et al. investigate how attentional patterns in humans and chimpanzees are influenced by predation. Utilising eye-tracking technology, they examined how features of potential predator and prey species affect attention, and how the gaze of the target animal modulates this phenomenon. This study is significant as our understanding of attentional mechanisms in response to predators and prey across different species remains limited. I would like to express my gratitude to the authors for their diligent efforts and for presenting a well-written and clear manuscript. I believe it is suitable for publication pending the below suggestions for improving the paper. Firstly, I believe that incorporating additional primate studies would complement the human research and comparative aspect of the manuscript. Secondly, I have some inquiries regarding the data processing and statistical analysis, which I will outline below. I hope these suggestions are constructive and beneficial for the authors.

Response :: We thank the reviewer for their constructive comments and efforts to make this a clearer, stronger manuscript.

ABSTRACT

**R2C2 ::** L29: It is not fully clear here what you mean with 'visual features'. Does this include the gaze orientation, or the low-level features which you discuss later on.

Response :: We have added text to clarify that this language was intended to indicate any visual features primates may perceive (ll 27-28).

**R2C3 ::** L32: “The gaze of the predators and prey”

Response :: This important omission is now corrected (ll 31).

INTRODUCTION

**R2C4 ::** - Because both human and non-human studies are addressed, it is important to mention this more explicitly. It is currently not always clear (e.g. L52)

Response :: We have addressed this ambiguity throughout the introduction (ll 50; 53; 57; 60).

**R2C5 ::** - From the first paragraph in the introduction it should be clear to the reader that attention is a limited cognitive capacity, and that this explains why attention is selected for relevant stimuli, e.g. animate objects.

Response :: We have edited the opening paragraph of the introduction to establish this fact of attention as a starting point (ll 44-47).

**R2C6 ::**  - Throughout the manuscript it is not really discussed that the prey is not necessarily a neutral animal, as (ancestral) humans hunt(ed) these species, making humans both a predator and a prey. Similar arguments can be made for chimpanzees.

Response :: We thank the reviewer for this point, and have added language to clarify what our use of impala images does and does not test (ll 175-180).

**R2C7 ::** - Within the introduction there is a mix between eye contact with conspecifics and heterospecifics for which we can expect different attentional and motivational systems. Barrett 2015 has written a chapter that explains this nicely: Barrett, H. C. (2015). Adaptations to predators and prey. The handbook of evolutionary psychology, 200-223. This chapter can be useful in general as they touch on the topic of forward-facing eyes of predators (p 208).

Response :: Thank you for this suggestion that has now been incorporated into the revised manuscript (ll 116-119).

**R2C8 ::** L67: You could strengthen your argument here by mentioning that it is likely that for most participants in these studies these are innate responses as they probably have never encountered such animals in real life (unless you have information about the exposure of the humans to lions).

Response :: We have added language to this effect (ll 71-73).

**R2C9 ::** L68-71: I think this sentence can be removed as it is focusing on conspecific interactions, which are fundamentally different from predator-prey interactions.

Response :: This is a reasonable point. After considerable reflection because our stimuli manipulate apparent threat in addition to predator/prey category membership, we prefer to retain the published work on attention to threatening faces for both humans and nonhuman primates.

**R2C10 ::** L73: I think there is a number of primate papers that are missing that would strengthen this argument, especially since you are comparing human and chimpanzees.

The following paper found evidence for attention biases in bonobos for leopards:

Laméris, D. W., Verspeek, J., Eens, M., & Stevens, J. M. G. (2022). Social and nonsocial stimuli alter the performance of bonobos during a pictorial emotional Stroop task. American Journal of Primatology, 84(2), e23356. <https://doi.org/10.1002/ajp.23356>

The following papers report attention biases for poisonous animals (e.g., snakes)

Masataka, N., Koda, H., Atsumi, T., Satoh, M., & Lipp, O. V. (2018).
 Preferential attentional engagement drives attentional bias to snakes in Japanese macaques (Macaca fuscata) and humans (Homo sapiens). Scientific Reports, 8(1), 17773. <https://doi.org/10.1038/s41598-018-36108-6>

Hopper, L.M., Allritz, M., Egelkamp, C. L., Huskisson, S.M., Jacobson, S. L., Leinwand, J. G., & Ross, S. R. (2021). A comparative perspective on three primate species’ responses to a pictorial emotional stroop task. Animals, 11(3), 588. <https://doi.org/10.3390/ani11030588>

Shibasaki, M., & Kawai, N. (2009). Rapid detection of snakes by japanese monkeys (Macaca fuscata): an evolutionarily predisposed visual system. Journal of Comparative Psychology, 123(2), 131–135. <https://doi.org/10.1037/a0015095>

Response :: We have incorporated these papers into the introduction (ll 66-71).

**R2C11 ::** L77: Replace 'new' with 'recent'

Response :: We have made this change (ll 79-80).

**R2C12 ::** L87-89: I feel Kano et al. (2015) is missing here. They report that bonobos, as compared to chimpanzees, pay more attention to eye regions.

Kano, F., Hirata, S., & Call, J. (2015). Social attention in the two species of pan: Bonobos make more eye contact than chimpanzees. PLoS ONE, 10(6). <https://doi.org/10.1371/journal.pone.0129684>

Response :: We have added this context (ll 93-95).

**R2C13 ::** L89-91: Please highlight that this is likely more true between conspecifics, whereas the current paper is focusing on heterospecifics

Response :: We have added this context, and indeed hope that our manuscript can shed light on how nonhuman great apes view heterospecific eyes (ll 91).

**R2C14 ::** L92: Please see Tomonaga & Imura (2015) DOI: 10.1038/srep11437

Response :: We have added this citation (ll 99).

**R2C15 ::** L97-100: I think it's debated whether the attention bias for snakes/spiders is due to their face. For example, in snakes the shape and presence of the scales drive responses (<https://doi.org/10.1111/psyp.12564> and Kawai, N., & Kawai, N. (2019). Searching for the Critical Features of Snakes. The Fear of Snakes: Evolutionary and Psychobiological Perspectives on Our Innate Fear, 121-153.) Please rephrase this sentence.

Response :: We have edited this sentence for clarity (ll 104-107).

**R2C16 ::** L137: "or other alternative hypotheses" This is unclear, what do you exactly mean?

Response :: We have removed this ambiguous language as part of a larger effort to clarify our hypotheses and predictions (ll 140-164). We did not make specific predictions for anticipated differences between gaze behaviors of humans and chimpanzees, and offer the learning and/or sociocultural determinants of any species differences only as [unlikely] alternatives to the evolutionarily-conserved attentional biases described in the introduction.

**R2C17 ::** L138: Please elaborate on other reasons why chimpanzees would not show similar attentional biases? There is literature on pareidolia effects in primates that can be useful to discuss whether or not you would expect similar results.

Response :: We hope that the larger effort to clarify our hypotheses and predictions makes clear that we did not predict, nor test for, differences in attentional biases across the species (ll 140-164). There are indeed good reasons to suspect evidence of evolutionarily-conserved attentional biases in both populations, and our intention was to seek evidence for these attentional biases in humans (Experiment 1) then in chimpanzees (Experiment 2) in a way that does not violate null hypothesis significance testing conventions. We have also added additional language to our citation of Taubert et al.’s rhesus macaque pareidolia research to better highlight this justification of existing research on conserved attentional biases in nonhuman primates (ll 101-103).

METHODS

**R2C18 ::** - Eye-tracking Analysis: I understand that for ape studies the trial duration is preferably shorter than for human studies. However, for a more fair comparison of the two species I would think you can make the argument to only analyse the first 3s of the human data. An extension of 5s is significantly long to capture additional attentional processes. I realise this might be a lot to request, and perhaps I am missing the rationale of why you looked at the full 8s human trials, but as you are comparing humans with chimpanzees I would expect a more similar study design.

Response :: Note that chimpanzees saw three repetitions of each image, an experimental manipulation intended to roughly control the total onscreen gaze time to each image across humans and chimpanzees. That chimpanzee exposure was broken up across these repetitions is indeed a difference that could affect gaze behaviors, albeit an aspect of the design that conforms with previous chimpanzee designs and with the constraints of working with these highly-enriched, socially-housed animals. Direct comparison of gaze mechanisms of humans and chimps was not a focus of our designs, and we instead intended to test our hypotheses about the effects of predator/prey features and eye direction, using the established norms for testing each species.

**R2C19 ::** L158: Please report whether students provided consent

Response :: We have added this language (ll 210-211).

**R2C20 ::** L174: It is not fully clear if both the Free-view task and Danger Rating task consisted of 96 images. Please clarify. Did the two tasks use the same images?
 I see now that you later discuss this at L199. Perhaps to avoid confusion you can mention this earlier?

Response :: We have clarified that both tasks used the same set of images in language at the start of the Procedure subsection (ll 223).

**R2C21 ::** L202-204: But did the human participants rate the lions are more dangerous than the impalas?

Response :: Yes, lions were rated as significantly more dangerous on a 5 point Likert scale (*M* = 3.55) than impala (*M* = 1.48). We have added text about these analyses to the revision (ll 249-250)

**R2C22 ::** L212-214: Is the original range of the two wild-born chimpanzees known? Where they actually savanna dwelling chimpanzees?

Response :: We have added language to the methods specifying that the wild-born chimpanzees were not savanna-dwelling (ll 256-257). As above we have revised our language throughout to make clear that although we recognize that there could be ecologically-informed hypotheses for chimpanzee behavior towards lions and impala, we mean only to test the uninformed responses of naive chimpanzees to new stimuli.

**R2C23 ::** L264-267: If a first trial was deemed successful, were subsequent repeated trials still included in the analysis? There could be a risk of habituation effects. This could be something you can additionally test to ensure whether or not this occurred.

Response :: All valid trials were analyzed, and we did not find evidence of habituation effects.

**R2C24 ::** L281: Please highlight that you used pictures of female impalas, as earlier mentioned in L149

Response :: We have added this language (ll 329).

**R2C25 ::** L281: I appreciate the author's efforts into finding this large number of different stimuli as it can be challenging to collect sufficient suitable stimuli. I think it would increase transparency if you mention whether you aimed to control for background features, or body orientation of the animals. E.g. for directed images the animal can be approaching the camera, facing the viewer, or can walk parallel to the camera but turn its head towards the camera (e.g. the two impala images in Figure 1). I'm aware that you partially corrected for this difference in ROI area in the processing, but it would benefit the reader if this information is provided.

Response :: We appreciate you bringing up this consideration. We have now added additional information about the stimuli noting that we did not precisely control for body orientation (for reasons you mention) and added information about background features (ll 332-335).

**R2C26 ::** L300: As you are analysing initial fixations it is really important that you report how the fixation cross proceeded. For the human study this is more controlled as the participants decided themselves when they continued, but for the chimpanzees this is not clearly reported. Was this manually done, after an Xms duration or when the chimpanzee was fixating at the cross. Eye-tracker systems vary in how this can be achieved. Additionally, did you verify whether the participants were in fact focusing on the fixation cross?

Response :: This is indeed an important consideration and we have added language describing this procedure for the chimpanzees (ll 300). Gaze behavior to both calibration and fixation points were highly precise around the designated points.

**R2C27 ::** Statistical analysis: I would like to ask for the rational to go for ANOVAs over mixed models. Did the data meet the assumptions for an ANOVA (homogeneity of variance, normality). I was under the impression that ANOVAs are typically not suitable for proportional data, especially when there are a lot of extreme values (0 or 100 in this case). Wouldn't a mixed model with an appropriate distribution be better? This way you would be able to control for number of trial repetitions for example.

Response :: An ANOVA is a conventional linear model for testing for differences among continuously-distributed (here, bounded at 0 and 100) data predicted by discrete experimental conditions. We did not identify any serious violations of ANOVA assumptions (e.g., Mauchley’s test of sphericity did not suggest this assumption was violated), which is now noted in the revision (ll 396-397). A generalized linear model could indeed match a likelihood function to the distributional character of our proportion data, incorporate additional predictors (that were not the target of our analyses), among other virtues. We have made our complete dataset available for interested parties to reanalyze, and are confident that the character of our inferences (e.g., high attention to directed gazes, eyes and lions) are not biased by the choice of analyses. The simple effects / contrasts comparing proportions within and across gaze types / ROIs / species are the main focus of our predictions, and these are not materially affected by the overall choice of model.

**R2C28 ::** L328: Please mention what these a priori contrasts were

Response :: We have added this language (ll 390-392).

RESULTS

**R2C29 ::** - In my opinion, the Results section would improve by clearly mentioning the model which is being discussed. So either, label the different models (model 1, model 2, model 3) in the "statistical analysis" and present the results in this way. Or perhaps better, present the results in terms of the predictions. Because you use one model for multiple predictions, this can create some confusion of what is actually being discussed.

Response :: This is indeed a meaningful improvement – we have added the Results subheading associated with each set of analyses described in the “Statistical Analysis” section (ll 378-388).

**R2C30 ::** - My understanding is that in a significant three-way interaction, looking at significant two-way interaction effects can be misleading, or incomplete, as the three-way interaction suggests that the two-way interaction depends on the third variable. Given that you report a significant three-way interaction for both humans and chimpanzees, I'm not fully convinced that the two-way interactions you report are meaningful. I'm aware that looking at two-way interactions can be meaningful in certain cases, but if you think this applies to your results I think it would be good practice to explicitly mention this. For example, you report that there is a difference in fixation proportion for the impala and lion eyes (L355-356), however, when looking at Figure 2 there appears a modulating effect of the gaze direction for the lion stimuli that possibly explains this effect. You discuss this in the lines after, but in reporting your results you jump between mentioning the three-way and two-way effects which is confusing. Given the presence of the three-way interaction I think it is more appropriate to report the significant effects and then focus on the insignificant contrasts within that three-way interaction, rather than the significant two-way interactions.

Response :: We apologize for any confusion and thank you for suggestions for how to clarify the results we report. The text of the Results section almost exclusively discusses the planned contrasts among the conditions related to our predictions for the exact reasons that you gave – the overall model and significant three-way interactions among animal type x ROI x gaze direction makes the network of relationships among these conditions impossible to discern using only the omnibus test. We are reluctant to focus on insignificant contrasts, given the *a priori* assumption of the null hypothesis in frequentist null hypothesis significance testing, but we have made many minor language edits to the Results to clarify what our tests revealed and added language to the Discussion acknowledging the limitation of using this zero-sum proportion measure that can produce some insidious modulation effects that you identified (ll 411-415).

**R2C31 ::** - Is there a reason why you did not directly test if the (initial) fixation proportion was associated with the saliency of the ROI? To me this would provide more reliable evidence that gaze patterns were not influenced by such low-level features.

Response :: The initial fixation proportions presented in section 3.2 and Fig 3 are principally to the eyes and head of the lion and impala images, regions that are not particularly salient in the saliency analyses presented in Fig 4 and Table 4. Initial fixation proportions were also influenced by the gaze direction of the lion or impala image in a way that saliency was not. More granular analyses are possible, and our dataset will be available for alternative approaches that are perhaps outside the scope of our questions.

DISCUSSION

**R2C32 ::** - Throughout the discussion I'm missing discussion that humans have arguably different relationships with lions/impalas than (captive) chimpanzees. For example, lions are typically considered charismatic species that are highly favoured to see in zoos or safaris. This is not to say that this necessarily drove your results (especially as you found very similar results between humans and chimpanzees), but I would like to see more discussion on how the difference in results between the species can be explained

Response :: We have added language to this effect to the Discussion (ll 516-520), a bookend to our acknowledgment in the introduction that differences among the patterns of gaze direction between chimpanzees and humans could be understood as the result of differences in the species’ previous experiences with images of lions and impala.

**R2C33 ::** - I'm missing a more detailed discussion into why the chimpanzees seemed to fixated more on the impala bodies. Could it be that you find similar results in humans when you focus on the first 3 seconds?

Response :: We have added additional speculation to the Discussion (ll 527-530), and none of our analyses suggested that differences in experimental design between the species affected the observed patterns of gaze behavior.

**R2C34 ::** L415: Please highlight again that this partly supports the first prediction (which included both humans and chimpanzees) as you could only test this for the chimpanzees. Additionally, perhaps you can make suggestions as how future studies can test this in a better way (i.e., avoiding ceiling effects as found with the human participants). E.g., showing impala and leopard pictures at the same time.

Response :: We have corrected that our first prediction was partially supported (ll 472) and incorporated that a future direction might better test this prediction with a preferential viewing design (ll 474-478).

**R2C35 ::** L422-424: Please highlight that gaze patterns for conspecifics can be different from those for heterospecifics. Guo et al. 2019 (<https://doi.org/10.1007/s00221-019-05574-3>) comes to mind that reports some work on this.

Response :: We have added this caveat and justifying citation (ll 484-486).

**R2C36 ::** L431: Please highlight that this is mostly for nonhuman heterospecific animals as there are multiple studies that report this when using conspecific stimuli.

Response :: This is indeed sharper language and we have added this clarification (ll 494).

**R2C37 ::** L433: For example, this statement is based on the two-way interaction whereas Figure 2 suggests a three-way interaction. Here you would need to report if the humans and chimpanzees also fixated more on the averted lion eyes than the averted impala eyes.

Response :: These statements should be understood as derived from the means, plots, and planned contrasts, rather than the omnibus test.

**R2C38 ::** L445-451: This paragraph is not very convincing in explaining why the saliency analyses show that bottom-up processing does not account for the gaze patterns. If this is mostly based on the fact that the differences in saliency do not align with the differences in gaze patterns, I do not think this is sufficient to exclude bottom-up processing

Response :: We have added language to moderate this point somewhat, as there could indeed be other low-level features (ll 509-511).

**R2C39 ::** L457-459: Kano 2009 (doi:10.1098/rspb.2008.1811) and Kano 2011 (DOI 10.1007/s10071-011-0422-5) can be useful here where they report that chimpanzees after faster fixation rates than humans

Response :: We have added these useful citations (ll 527).

**R2C40 ::** L459: Please change 'is' to 'could be'

Response :: We have made this change (ll 541-542).

**R2C41 ::** L466-468: This is just a little confusing because you look at both 3- and 2-way interactions which result in somewhat contrasting results

Response :: Because of the 3-way interactions among animal type, ROI, and gaze direction our hypotheses were tested using the planned comparisons presented in Table 4.

REFERENCES

**R2C42 ::** - Kano et al. 2018 (L429) is missing from the reference list - Please add the year in Yorzinski L669

Response :: We apologize for the errors, which have now been corrected.
