# Supplementary material for: Predator gaze captures both human and chimpanzee attention
Source: PLoS One. 2024 Nov 21;19(11):e0311673. doi: 10.1371/journal.pone.0311673 (PMC11581262; doi:10.1371/journal.pone.0311673)
Supplement: Attachment — Submitted filename: Response to Reviewers.docx [file pone.0311673.s010.docx]

Reviewer comments

I would like to thank the authors for responding to my previous comments. I appreciate some of the adjustments made by the authors which already improved the quality of the manuscript. However, there remain several points where I believe the authors' responses were not entirely adequate which I list below.

MAJOR COMMENTS

RC1 :: I continue to encounter major concerns with the difference in study designs for the human and chimpanzee study. While I acknowledge the authors’ clarification that it was not the intention to formally test species differences, many sentences throughout the manuscript suggest a comparability between the human and chimpanzee findings:

L36-38: “The striking similarities between the gaze patterns of humans and chimpanzees provide additional evidence that attentional processing of two-facing eyes is evolutionarily conserved across primates.”.

L141-144: “Attentional biases for predators over prey in modern humans were likely shaped by the same sources of natural selection as those experienced by chimpanzees and as a result patterns of chimpanzee attention should reflect the same (or similar attentional biases)”. You need to statistically test for this. Simply comparing patterns is not scientifically sound.

L486-488: “These chimpanzee results cohere with prior research suggesting that apes attend to eyes of conspecifics and heterospecifics but do so less often than human participants (Kano et al., 2018).”

L514-530: This entire paragraph compares the chimpanzee results to the human results. I don’t see how this is justified if you used a different study design and therefore potentially investigated two completely different things.

Response :: We have removed the specific sentences of major concern. We have also, in response to several of the constructive comments below, substantially restructured the manuscript to more completely disambiguate the two experiments given the differences in design and methodology.

RC2 :: Linked to my previous point, the authors’ response fails to address why a similar study design wasn’t initially employed, or why they do not want to analyze only the first 3s of the human data, as previously suggested. The authors replied stating that chimpanzees saw three repetitions of each image, but the text mentions that each image was shown four times. Moreover, the argument that by repeating the trials was intended to roughly control the total screen time does not address my concern. The discrepancy between eight consecutive seconds and 3 sets of 3 seconds each is substantial, potentially leading to disparate gaze patterns and undermining the comparability of human and chimpanzee trials (e.g., it is unlikely that the chimpanzees would pick up their gaze patterns where they left in the previous trial). I strongly recommend the authors to reconsider only analyzing the first 3s of the human trials so that a comparison can actually be made. I am aware that the authors state that a human-chimpanzee comparison was not a study aim, but above I mention a couple of places in the paper where this is not clear and where readers can easily misinterpret this. In this way, the title and abstract are already not transparent as there is no mention of this difference in study design. Not even in the discussion is this limitation of the study mentioned.. Similarly for the predictions, I appreciate that the authors included the sentence stating that they did not make specific predictions about divergent patterns, but you actually ran two experiments with different study designs. This needs to be mentioned and you need to make specific predictions about this. Alternatively, the manuscript needs to be restructured in a way that it becomes clear that you tested the humans and chimpanzees on different tasks.

Response :: We have followed the advice to restructure the manuscript in a way that more clearly emphasizes the differences of procedure, analyses, and conclusions between the human and chimpanzee designs. In the general discussion we again emphasize these differences alongside the conservative inference that in both humans and chimpanzees we observed evidence of the biases to lions and to their directed gaze that we predicted (ln 575-590). We intended 3 repetitions of a stimulus to indicate the four total exposures described in the manuscript, but apologize for the unclear language.

RC3 :: I thank the authors for their additional explanation regarding their statistical approach. I'm, however, still not convinced about including a three-way interaction but focusing on the significant two-way interactions (because these were planned contrasts). Such a selective approach may be perceived as cherry-picking, as it disregards other potential effects that may exist within the data. It also raises the question why, for example, the comparison between lion*eye*direct versus lion*body*direct is not made. From the graph it suggests that this effect is there and, to me, would give much stronger evidence for an attention bias for the eyes of a predator, incorporating your reasoning that the direction of the face is an important modulating factor. By ignoring the significant three-way interaction you're missing out on reporting results.

In L412 for example, you find a significant three-way interaction, indicating that the fixation proportion is influenced by an interplay between those three factors. But here, by looking at the two-way interaction your possibly just ignoring a possible significant effect between lion*head*direct and lion*body*direct. Alternatively, in the next sentence you focus on the significant stimulus type*ROI effect and report that humans and chimpanzees fixated more on lion eyes than the impala eyes, but from the graph it is obvious that gaze direction modulates this effect (for the lions).

Response :: We appreciate the additional notes on statistical interpretation of the results. The full matrix of three-way post hoc comparisons are briefly discussed in new sections added to the results for Experiment 1 (ll 358-370) and Experiment 2 (ll 539-545), visualized in new supplements S1 and S7, and reported explicitly in supplements S2 and S8. We attempted throughout to tailor our analyses to the specific predictions we made about anticipated patterns of gaze data. Planned contrasts were targeted to our specific statistical predictions about anticipated differences that may emerge from the hypothesized attentional biases that are the subject of the manuscript.

RC4 :: Additionally, I find the authors' response as to why the saliency was not directly tested against the (initial) fixation proportion unsatisfactory. Mentioning that the data will be made available for alternative approaches that are outside the scope of your questions is not true, and truthfully suggests reluctance from the authors to consider changes. It’s essential to note that the current methodology precludes conclusive remarks on the impact of saliency on gaze patterns, as highlighted in specific sections of the manuscript (e.g., L504-508, L532—535). Given that the effect of saliency on AOIs is one of your main predictions, it is essential to use proper statistical methods to actually make any conclusions. I very much appreciate the inclusion of the saliency maps in this paper which is exemplary for other studies, but if the authors do not want to properly test this, I would suggest removing this component from the study. I reiterate the recommendation for using mixed models which would allow for incorporating the saliency values as a control variable or covariate.

Response :: The salience analyses have been reworked to more directly test the predictive validity of salience values on initial fixation proportions using a framework that tests the salience at the fixated region to two chance-based estimates (ll 280-296; 347-356; 533-537). Salience maps do not predict the pattern of gaze behaviors that were the subject of our design, analyses, and conclusions.

RC5 :: I agree with Reviewer 1 that within the primate literature total fixation duration is more common. While the authors reply that proportion of fixations aligns with previous human work, this could similarly be applied to primate studies, so it is not clear why this approach was favored over the other. I appreciate the addition of figure S4 showing these data, and the authors state that no new effects emerged from the datasets with normalized total fixation duration is used as dependent variable. However, no statistics are provided or indicated in the figure. Again, simply comparing patterns between graph is not sufficient to make such conclusions. Even then, just by scanning figure S4 and comparing with figure 2B, it seems clear that the two do not follow the same pattern (but then again, we would need statistics to know this, just like with the saliency results). It is also unclear why only chimpanzee data are shown and not human data.

Response :: We clarify the relative merits of using area-normalized fixation proportions for designs of this kind in the Eye-tracking analysis text (ln 264-272). We have added ANOVA and planned comparisons analyses on total fixation duration to figure S4 to parallel those of the main analysis of the manuscript. Parallel analyses with the human data are unfortunately not possible using as the software did not record fixation times to background or off-screen fixations. As with the planned contrasts above, we favor a selective approach that is targeted to the specific predictions we made about anticipated patterns of gaze data using the dependent variable we intended to base our analyses on.

MINOR COMMENTS

RC6 :: L68-69: I would add the species for each of the references.

Response :: We have made this change (ln 63-67)

RC7 :: L71: Replace ‘lions’ with 'animals' or something alike as you don't only mention lions.

Response :: We have made this change (ln 67-70)

RC8 :: L73-75: I would move this to the next paragraph where you actually introduce that faces are relevant.

Response :: We have made this change (ln 79-81)

RC9 :: L95: You start the sentence with “One study found…” but you actually refer to multiple studies, please revise.

Response :: We have made this change (ln 94-96)

RC10 :: L115: I would add the argument of the pareidolia effect at the end of this sentence to highlight that this is such a strong tendency that it extends beyond natural faces.

Response :: We have made this change (ln 112-114)

RC11 :: L122: Senju et al. 2011 is an interesting paper here: <https://doi.org/10.1080/13506280444000157>

Response :: One of our favorites, cited ln 95-96

RC12 :: L182: Rephrase: “and the face being directed or averted”

Response :: We have made this change (ln 172-174)

RC13 :: L376: “Section 3.1.1”

Response :: We have added “Section” to each leading reference throughout the manuscript.

RC14 :: L388: "stimulus species", or something to highlight it's about the lion and impala.

Response :: We have changed stimulus type to “stimulus species” throughout the manuscript

RC15 :: L410: If you used a linear mixed-effects model, I assume you included random effects. What were these?

Response :: These salience models are no longer a part of the manuscript in favor of the new approach to salience analyses described above.

RC16 :: Table 2: Having a second look at the table, the comparison Background direct vs. Background averted is missing.

Response :: The area-normalized fixation proportions are normalized such that the proportions sum to one, allowing for only 1 unique comparison in a comparison of only animals vs backgrounds. This aside, Table 2 is omitted from the rearranged manuscript since only the chimpanzee data were interpretable.

RC17 :: L475-479: But you did not test the difference between for example impala head and body, so you cannot conclude this (or these statistics are at least not reported in the text and tables). At the same time, this was tested for in the saliency analyses. This raises concerns regarding the clarity and coherence of the testing procedures, potentially impeding readers' understanding of the tested variables and their corresponding results. Given this inconsistency, readers may find it challenging to track and interpret the outcomes of the various analyses conducted.

Response :: Table 4 is incidentally not needed in the revised manuscript due to the reconfiguration of salience analyses to use Mann-Whitney tests to more directly test the relationship between initial fixations and salience. The tests targeted to our specific hypotheses should be consistent throughout the manuscript, and all unplanned comparisons are available in supplemental materials.

RC18 :: L484-486: Again, not listed in the tables.

Response :: We would be happy to address this comment with additional context.
