# Supplementary material for: Predator gaze captures both human and chimpanzee attention
Source: PLoS One. 2024 Nov 21;19(11):e0311673. doi: 10.1371/journal.pone.0311673 (PMC11581262; doi:10.1371/journal.pone.0311673)
Supplement: Attachment — Submitted filename: Response to Reviewers.docx [file pone.0311673.s011.docx]

Reviewer comments

Reviewer #2: Dear authors,

RC1: Thank you for addressing the comments and suggestions provided in the previous round of review. I have reviewed the revised version of your manuscript and am pleased to note the improvements made. The manuscript is now significantly clearer and more transparent, making it much easier to read and understand. The adjustments you have made have strengthened the overall quality of the paper. I appreciate the effort you have put into refining the content, particularly in the areas of data presentation, separating the two experiments and interpretation of the results. Overall, I am satisfied with the revisions and believe the manuscript is now in a much better state for publication. I have no further major concerns, and I commend you on the work done to enhance the clarity and rigor of the research presented.

Response: We thank the reviewer for their constructive comments throughout

Minor comments:
L113-115: Redundant? Has been discussed earlier. Maybe can be integrated.

Response: We have moved these citations about human and other primates’ apparent bias to faces a few lines higher (ll 109-112) to sit after “These findings suggest that, as with human faces, the bias to attend to nonhuman animal faces is especially strong when direction of gaze is aimed toward the observer.”

L327: I assume you used corrected p-values? I would suggest reported what significance values were handled to enhance transparency to the readers. Perhaps I missed it.

Response: Bonferroni-correct *p* values were used for all inferences over the *post hoc* contrasts (ll 365, 545)

L354: I don’t know if the journal follows APA-style, but there the z value is reported before the p value.

Response: We have rearranged these reports to position *z* values before *p* (ll 357-359, ll 537-538).

L495: I know that the human participants rarely gazed at the backgrounds, but for consistency I think it'd be better to provide this information for Exp1 as well.

Response: We have added specific means, standard deviations, and confidence intervals to the Experiment 1 Animals vs Backgrounds sections as well (ll 319-321).

L575: I would be careful calling the designs similar.

Response: We have removed this language.
